# KIR repertory in patients with hematopoietic diseases and healthy family members

**DOI:** 10.1186/s12878-016-0064-6

**Published:** 2016-09-29

**Authors:** Daniele Kazue Sugioka, Carlos Eduardo Ibaldo Gonçalves, Maria da Graça Bicalho

**Affiliations:** Departamento de Genética, Laboratório de Imunogenética e Histocompatibilidade (LIGH), Universidade Federal do Paraná, R. Cel. Francisco H. dos Santos S/N, Centro Politécnico – Jardim das Américas, CEP 81.530.990, Curitiba, PR CP 19071 Brazil

**Keywords:** KIR, HLA, Leukemia

## Abstract

**Background:**

Since the discovery of specific histocompatibility, literature has associated genes involved in the immune response, like the Human Leucocyte Antigen (HLA), with a better prognosis in transplantation. However, other non-HLA genes may also influence the immune process, such as the genes encoding the immunoglobulin-like receptors of natural killer cells (KIRs). The discovery that NK cell KIR receptors interact with conservative epitopes (C1, C2, Bw4) presented in HLA class I molecules that are genetically polymorphic, also observed in KIR genes, led to the investigation of the relevance of the KIR system to hematopoietic stem cell transplant. The cure of patients with leukemias and other hematological malignancies after bone marrow transplantation (BMT) has been attributed in part to the ability of the donor immune cells, present in the graft, to recognize and eliminate neoplastic cells of the patient. The cytotoxic activity of NK cells is mediated by the absence of HLA class I-specific ligands on the target cell surface to inhibitory KIR receptors (hypothesis of “missing-self”).

**Methods:**

We analyzed, by PCR typing-SSOP technique, the presence or absence of 16 KIR genes and haplotypes of 39 patients with hematopoietic disorders and 136 healthy individuals from Paraná State. The comparisons made between the patient and control group were performed using χ^2^ test or Fisher exact test (bilateral *p*-value), as appropriated. Significance level was considered when *p*-value ≤ 0.05.

**Results:**

Framework genes *KIR3DL3, KIR3DP1, KIR2DL4* and *KIR3DL2* were positive in all samples. The comparison between KIR repertoire of patients and healthy individuals revealed significant differences (*p* < 0.05) in inhibitors genes *KIR2DL2* (*p* = 0.0005) and *KIR2DL5* (*p* = 0.0067) and activating genes *KIR2DS1* (*p* = 0.0013), *KIR2DS2* (*p* = 0.0038), *KIR2DS3* (*p* = 0.0153) that are more frequent in controls than in patients. The *KIR2DS3* was significantly more frequent (*p* = 0.0031) in patients with acute myeloid leukemia (AML) when compared to patients with acute lymphoblastic leukemia (ALL). We observed a higher frequency of haplotype A (59 %) in the patients.

**Conclusion:**

Our data suggests that susceptibility to leukemia can be influenced, at least, partly byKIR receptors.

**Electronic supplementary material:**

The online version of this article (doi:10.1186/s12878-016-0064-6) contains supplementary material, which is available to authorized users.

## Background

Natural killer (NK) cells play a pivotal role in innate immunity providing immediate protection against infections as well as in the early steps of neoplastic cellular transformation [1]. The antileukemic role of Natural Killer (NK) cells has been brought into focus in recent years.

Killer cell immunoglobulin-like receptors (KIRs) interactions with their ligands regulate the cytotoxicity activity of NK cells. HLA-Cw is the primary ligand for a significant number of inhibitory KIRs. HLA-Cw allotypes are categorized into C1 and C2 groups based on a polymorphism at residue 80 in the HLA-Cw molecule. Inhibitory *KIR2DL2* and *KIR2DL3* are specific for the C1 ligand group, and inhibitory *KIR2DL1* is specific for the C2 ligand group. The inhibitory *KIR3DL1* receptor is specific for HLA molecules with the HLA-Bw4 epitope (HLA-B, HLA-A3 e HLA-A11) [2]. When inhibitory KIRs encounter self-HLA class I ligands on target cells, they signal inhibition and establish tolerance.

*KIR* genotypes are organized into two main broad haplotypes termed A and B, according to KIR inhibitorys and activators genes content. Both A and B haplotype share four framework genes: *KIR2DL4, KIR3DL2, KIR3DL3* and *KIR3DP1*. Haplotype A gene organization includes up to eight genes, those of the framework content together with *KIR2DL1*, *KIR2DL3, KIR2DS4* and *KIR3DL1*. The activating *KIR* genes, *KIR2DS1, KIR2DS2, KIR2DS3, KIR2DS5,* and *KIR3DS1,* as well as the genes encoding inhibitory *KIRs, KIR2DL5A/B and KIR2DL2,* are the principal representants of the Group B haplotypes [3].

The “missing-self” concept presented by Kärre and colleagues in the 1980s, paved the way for the understanding of NK-derived allorecognition mechanism [1, 4]. In brief, NK cells, through the expressing of cognate inhibitory receptor learn to detect and kill cells with reduced or “missing” expression of “self” MHC class I ligands [5].

Since the early 70s hematopoietic progenitor cells transplantation from different sources, have been used as a therapeutic alternative for a broad spectrum of cancers and hematological diseases [6].

The past 10 years have witnessed dramatic progress in our understanding of possible exploitation of NK cells in cancer therapy. Haplo-Hematopoietic Stem Cell Transplantation (haplo-HSCT) outcoming showed a positive effect related to NK cells in adults with AML and also in children with high-risk ALL [7, 8]. The increased activity of NK cells after transplanting, even when the donor and recipient are HLA- identical, suggest that the cytotoxicity of these receptor cells from the donor can be an additional component previously unrecognized in the rejection process.

Several studies indicate an association with disease role in interactions between these KIRs and HLA loci and infectious diseases, autoimmune/inflammatory diseases, cancer and reproduction [9].

Only a few studies have investigated the association between the genetic diversity of activating and inhibitory KIR genes in humans and the susceptibility and resistance to leukemia [10]. Some results pointed out association between a group of activating and inhibitory KIR genes with relapse, overall survival and relative risk [11].

Some results pointed out the association between a group of activating and inhibitory KIR genes with relapse, overall survival and relative risk [11].

More studies on KIR allelic diversity are needed in order to clarify the role of NK cells in hematopoietic diseases. Among the three already well- known gene families which encode for NK cell receptors, we aimed to characterize the *KIR* genes repertoire in patients from Paraná State with hematopoietic disorders.

## Methods

### Samples

The sample consisted of 39 patients (as shown in Table 1) with HSCT indication from the Erasto Gaertner Hospital (Curitiba, Paraná State, Brazil) and 136 healthy family members from LIGH-UFPR (Laboratory of Immunogenetics and Histocompatibility, Federal University of Paraná) database, in the period of July 2007 to May 2008 to carry out pre-transplant histocompatibility testing. All participants signed an informed consent document. The study was approved by the Ethics Committee from UFPR-CEP-HC number 037ext.019/2001-07.

**Table 1:** General characteristics of patients (*N* = 39)

			Gender		Age		Ethnic group		
Disease	N	(%)	M	F	Average	Std. Dev.	White	Mulatto	Black
Bone Marrow Aplasia	1	0.03	0	1	30.0	0.00	1	0	0
Paroxysmal Nocturna Hemoglobinuria	2	0.05	2	0	31.5	2.10	2	0	0
NK Leukemia Cells	1	0.03	1	0	13.0	0.00	1	0	0
Non Hodgkin Lymphoma - Follicular	1	0.03	0	1	39.0	0.00	1	0	0
Acute Lymphoid Leukemia	13	0.33	7	6	15.2	16.70	11	2	0
Acute Myeloid Leukemia	11	0.28	7	4	35.7	17.80	3	0	0
Chronic Myeloid Leukemia	3	0.08	3	0	31.7	9.60	1	0	0
Myelodysplasia	1	0.03	1	0	23.0	0.00	2	0	0
Myelofibrosis	2	0.05	1	1	50.5	12.00	1	0	0
Multiple Myeloma	1	0.03	1	0	40.0	0.00	1	1	0
MyelodysplasticMyeloproliferativeSyndrome	2	0.05	1	1	42.0	1.40	10	1	0
Not Informed	1	0.03	1	0	NI	NI	1	0	0
Total	39	1	25	14			35	4	0

*M* male, *F* female, *Std. Dev.* standard deviation; IBGE ethnic classification

### Extraction of genomic DNA

Two tubes with 10 milliliters (10 ml) of peripheral blood were collected from each individual by venous puncture into sterile tubes containing EDTA vacutainer type. These samples were centrifuged to obtain leucocyte layer from which the DNA was extracted by salting-out technique [12]. The DNA concentration of the samples was measured by reading optical density, using the spectrophotometer Gene Quantpro RNA/DNA calculator.

### Typing of *KIR* genes

The *KIR* gene typing was performed by PCR-SSOP (Polymerase Chain Reaction - Sequence Specific Oligonucleotide Probes), amplifying the exons 3, 5 and 7–9, using the kit “Labtype*KIR* SSO Genotyping Test” (One Lambda Inc). The data analysis was performed using the HLA VISUAL version 2.0 software (One Lamda Inc.) that analyzes the combinations of probes in the microbeads detected by the instrument and consults an internal database that suggest what are the loci present.

### Statistical analysis

Phenothypic frequencies regarding presence/absence of *KIR* genes for all samples (patient group and healthy control group) along with the haplotype frequencies were obtained by direct counting.

The frequencies of the 16 *KIR* genes obtained for ALL and AML patients group were compared using the χ^2^ test, and the comparisons made between the patient and control group were performed using Fisher exact test (bilateral *p*-value), with the aid of BioEstat 5.0 software. When the sample size of the analyzed group was very small (less than 10), which can decrease the accuracy of the test χ^2^, Yates correction was applied. The frequencies of haplotypes A and B were obtained by direct counting. The comparisons made between patient and controls were performed using χ^2^test. Significance level was considered when *p*-value ≤ 0.05.

## Results

The epidemiology of the 39 patients with HSCT indication was analyzed according to disease, gender, age and ethnic/racial group (Table 1). Age analysis of the patients (*N* = 39) indicated that 64 % of the patients were male (25) and 36 % were female (14). The average age of the patients was 28.50 + 17.74 years. According to the racial group suggested by the Brazilian Institute of Geography and Statistics (IBGE), 89 % of patients were classified as White and 11 % Mulattos (mixed-descendent of White *vs* Black).

The frequencies of the presence/absence of 16 KIR genes in the patients are presented in Table 2. These data were compared with 136 healthy family members and it was observed that the ***KIR2DL2*** (*p* = 0.0005); ***KIR2DL5*** (*p* = 0.0067); ***KIR2DS1*** (*p* = 0.0013); ***KIR2DS2*** (*p* = 0.0038) and ***KIR2DS3*** genes (*p* = 0.0153) were statistically more frequent in the healthy individuals than in the patients as can be seen in Table 3. The comparison between patients with acute lymphoid leukemia (ALL) and acute myeloid leukemia (AML) showed that the ***KIR2DS3*** gene (*p* = 0.0013) was more frequent in AML patients (Table 4).

**Table 2:** Distribution of KIR genes frequenciesin patients (*N* = 39)

		*KIR* genes															
		2DL1	2DL2	2DL3	**2DL4**	2DL5	2DP1	2DS1	2DS2	2DS3	2DS4	2DS5	3DL1	**3DL2**	**3DL3**	**3DP1**	3DS1
Patients	absolutefrequency	35	9	36	39	11	37	6	12	5	36	11	37	39	39	39	10
*N* = 39	relativefrequency	0,90	0,23	0,92	1,00	0,28	0,95	0,15	0,31	0,13	0,92	0,28	0,95	1,00	1,00	1,00	0,26
	genefrequency	0,68	0,12	0,72	1,00	0,15	0,77	0,08	0,17	0,07	0,72	0,15	0,77	1,00	1,00	1,00	0,14
BoneMarrow Aplasia	absolutefrequency	1	0	1	1	1	1	1	0	0	1	1	0	1	1	1	1
*N* = 1	relativefrequency	1,00	0,00	1,00	1,00	1,00	1,00	1,00	0,00	0,00	1,00	1,00	0,00	1,00	1,00	1,00	1,00
	genefrequency	1,00	0,00	1,00	1,00	1,00	1,00	1,00	0,00	0,00	1,00	1,00	0,00	1,00	1,00	1,00	1,00
ParoxysmalNocturnaHemoglobinuria	absolutefrequency	2	0	2	2	0	2	0	1	0	2	0	2	2	2	2	0
*N* = 2	relativefrequency	1,00	0,00	1,00	1,00	0,00	1,00	0,00	0,50	0,00	1,00	0,00	1,00	1,00	1,00	1,00	0,00
	genefrequency	1,00	0,00	1,00	1,00	0,00	1,00	0,00	0,29	0,00	1,00	0,00	1,00	1,00	1,00	1,00	0,00
NK LeukemiaCells	absolutefrequency	1	0	1	1	0	1	0	0	0	1	0	1	1	1	1	0
*N* = 1	relativefrequency	1,00	0,00	1,00	1,00	0,00	1,00	0,00	0,00	0,00	1,00	0,00	1,00	1,00	1,00	1,00	0,00
	genefrequency	1,00	0,00	1,00	1,00	0,00	1,00	0,00	0,00	0,00	1,00	0,00	1,00	1,00	1,00	1,00	0,00
Non Hodgkin Lymphoma - Follicular	absolutefrequency	1	0	1	1	0	1	0	0	0	1	0	1	1	1	1	0
*N* = 1	relativefrequency	1,00	0,00	1,00	1,00	0,00	1,00	0,00	0,00	0,00	1,00	0,00	1,00	1,00	1,00	1,00	0,00
	genefrequency	1,00	0,00	1,00	1,00	0,00	1,00	0,00	0,00	0,00	1,00	0,00	1,00	1,00	1,00	1,00	0,00
AcuteLymphoidLeukemia	absolutefrequency	12	3	12	13	4	13	2	3	2	12	5	13	13	13	13	2
*N* = 13	relativefrequency	0,92	0,23	0,92	1,00	0,31	1,00	0,15	0,23	0,15	0,92	0,38	1,00	1,00	1,00	1,00	0,15
	genefrequency	0,72	0,12	0,72	1,00	0,17	1,00	0,08	0,12	0,08	0,72	0,22	1,00	1,00	1,00	1,00	0,08
AcuteMyeloidLeukemia	absolutefrequency	8	3	9	11	4	9	2	5	2	9	3	10	11	11	11	5
*N* = 11	relativefrequency	0,73	0,27	0,82	1,00	0,36	0,82	0,18	0,45	0,18	0,82	0,27	0,91	1,00	1,00	1,00	0,45
	genefrequency	0,48	0,15	0,57	1,00	0,20	0,57	0,10	0,26	0,10	0,57	0,15	0,70	1,00	1,00	1,00	0,26
ChronicMyeloidLeukemia	absolutefrequency	3	0	3	3	0	3	0	0	0	3	1	3	3	3	3	0
*N* = 3	relativefrequency	1,00	0,00	1,00	1,00	0,00	1,00	0,00	0,00	0,00	1,00	0,33	1,00	1,00	1,00	1,00	0,00
	genefrequency	1,00	0,00	1,00	1,00	0,00	1,00	0,00	0,00	0,00	1,00	0,18	1,00	1,00	1,00	1,00	0,00
Myelodysplasia	absolutefrequency	1	1	1	1	1	1	1	1	0	1	1	1	1	1	1	1
*N* = 1	relativefrequency	1,00	1,00	1,00	1,00	1,00	1,00	1,00	1,00	0,00	1,00	1,00	1,00	1,00	1,00	1,00	1,00
	genefrequency	1,00	1,00	1,00	1,00	1,00	1,00	1,00	1,00	0,00	1,00	1,00	1,00	1,00	1,00	1,00	1,00
Myelofibrosis	absolutefrequency	2	0	2	2	0	2	0	0	0	2	0	2	2	2	2	0
*N* = 2	relativefrequency	1,00	0,00	1,00	1,00	0,00	1,00	0,00	0,00	0,00	1,00	0,00	1,00	1,00	1,00	1,00	0,00
	genefrequency	1,00	0,00	1,00	1,00	0,00	1,00	0,00	0,00	0,00	1,00	0,00	1,00	1,00	1,00	1,00	0,00
MultipleMyeloma	absolutefrequency	1	1	1	1	0	1	0	1	0	1	0	1	1	1	1	0
*N* = 1	relativefrequency	1,00	1,00	1,00	1,00	0,00	1,00	0,00	1,00	0,00	1,00	0,00	1,00	1,00	1,00	1,00	0,00
	genefrequency	1,00	1,00	1,00	1,00	0,00	1,00	0,00	1,00	0,00	1,00	0,00	1,00	1,00	1,00	1,00	0,00
MyelodysplasticMyeloproliferativeSyndrome	absolutefrequency	2	0	2	2	0	2	0	0	0	2	0	2	2	2	2	1
*N* = 2	relativefrequency	1,00	0,00	1,00	1,00	0,00	1,00	0,00	0,00	0,00	1,00	0,00	1,00	1,00	1,00	1,00	0,50
	genefrequency	1,00	0,00	1,00	1,00	0,00	1,00	0,00	0,00	0,00	1,00	0,00	1,00	1,00	1,00	1,00	0,29
NotInformed	absolutefrequency	1	1	1	1	1	1	0	1	1	1	0	1	1	1	1	0
*N* = 1	relativefrequency	1,00	1,00	1,00	1,00	1,00	1,00	0,00	1,00	1,00	1,00	0,00	1,00	1,00	1,00	1,00	0,00
	genefrequency	1,00	1,00	1,00	1,00	1,00	1,00	0,00	1,00	1,00	1,00	0,00	1,00	1,00	1,00	1,00	0,00

Genes in bold are those genes called “framewok genes

**Table 3:** Frequency of each KIR gene in patients (*N* = 39) and controls (*N* = 136)

	*KIR* gene	Patient	Control	*p*-value
		(*n* = 39)	(*n* = 136)	
Framework	*KIR2DL4*	39 (100 %)	136 (100 %)	1.0000
	*KIR3DL2*	39 (100 %)	136 (100 %)	1.0000
	*KIR3DL3*	39 (100 %)	136 (100 %)	1.0000
	*KIR3DP1*	39 (100 %)	136 (100 %)	1.0000
Haplotype A	*KIR2DS4*	36 (92 %)	129 (95 %)	0.6936
	*KIR2DL1*	35 (90 %)	130 (96 %)	0.2329
	*KIR2DL3*	36 (92 %)	118 (87 %)	0.4173
	*KIR3DL1*	37 (95 %)	129 (95 %)	1.0000
Haplotype B	***KIR2DS1***	6 (15 %)	59 (43 %)	**0.0013 (** ^a^ **)**
	***KIR2DS2***	12 (31 %)	78 (57 %)	**0.0038 (** ^a^ **)**
	***KIR2DS3***	5 (13 %)	45 (33 %)	**0.0153 (** ^a^ **)**
	*KIR2DS5*	11 (28 %)	45 (33 %)	0.6976
	*KIR3DS1*	10 (26 %)	53 (39 %)	0.1356
	***KIR2DL2***	9 (23 %)	75 (55 %)	**0.0005 (** ^a^ **)**
	***KIR2DL5***	11 (28 %)	72 (53 %)	**0.0067 (** ^a^ **)**
Haplotype A/B	*KIR2DP1*	37 (95 %)	131 (96 %)	0.6465

Genes in bold showed significance for the statistical test (^a^)

**Table 4:** Frequency of each KIR gene from patients with ALL (*N* = 12) and AML (*N* = 11)

		All	AML	*p*-value
	*KIR* GENE	(*n* = 12)	(*n* = 11)	
Framework	*KIR2DL4*	12 (100 %)	11 (100 %)	1.0000
	*KIR3DL2*	12 (100 %)	11 (100 %)	1.0000
	*KIR3DL3*	12 (100 %)	11 (100 %)	1.0000
	*KIR3DP1*	12 (100 %)	11 (100 %)	1.0000
Haplotype A	*KIR2DS4*	11 (92 %)	9 (82 %)	0.5921
	*KIR2DL1*	11 (92 %)	8 (73 %)	0.3168
	*KIR2DL3*	11 (92 %)	9 (82 %)	0.5901
	*KIR3DL1*	12 (100 %)	10 (91 %)	0.4783
Haplotype B	*KIR2DS1*	10 (83 %)	9 (82 %)	1.0000
	*KIR2DS2*	3 (25 %)	3 (27 %)	1.0000
	***KIR2DS3***	2 (17 %)	9 (82 %)	**0.0031 (** ^a^ **)**
	*KIR2DS5*	5 (42 %)	3 (27 %)	0.6668
	*KIR3DS1*	2 (17 %)	5 (45 %)	0.1930
	*KIR2DL2*	3 (25 %)	3 (27 %)	1.0000
	*KIR2DL5*	4 (33 %)	4 (36 %)	1.0000
Haplotype A/B	*KIR2DP1*	12 (100 %)	9 (82 %)	0.2174

Genes in bold showed significance for the statistical test (^a^)

We also observed a higher frequency of haplotype A in patients (59 % of haplotype A and 41 % of haplotype B).

## Discussion

Fourteen KIR genes plus two pseudogenes are joined in the leukocyte receptor complex (LCR) on chromosome 19q13.4 and display a high degree of genetic diversity concerning gene content and allelic polymorphism [13]. This genomic structure drives to non-allelic homologous recombination events, which potentially generates considerable genetic diversity in KIR gene repertoire among individuals and populations [14].

*HLA* and *KIR* gene clusters are functionally linked but segregate independently creating a genetic diversity that could have a different impact on transplantation outcome. Hence, studies regarding KIR and HLA genes in different populations can provide valuable information to several scientific fields [15, 16].

In our study, we evaluated the repertoire of *KIR* genes in patients with hematopoietic disorders. Overall, the frequencies of the presence/absence of *KIR* genes were similar to the frequencies observed in European populations, which would be expected considering the predominance of White Euro-descendants in southern Brazil.

The framework genes *KIR3DL3, KIR3DP1, KIR2DL4* and *KIR3DL2* were observed in all samples, in accordance to their presence in all known KIR haplotypes. The remaining inhibitorys and activators *KIR* genes showed frequencies that varied between individuals. The distribution of *KIR* gene frequencies among patients showed low frequencies as follows: *KIR2DL2* (23 %), *KIR2DL5* (28 %), *KIR2DS1* (15 %), *KIR2DS3* (13 %), *KIR2DS5* (28 %) and *KIR3DS1* (26 %). Despite the small sample size, the results are in agreement with those observed for populations of Caucasian origin. The *KIR* genes that showed higher frequencies in the patient group were: *KIR2DL1* (90 %), *KIR2DL3* (92 %), *KIR2DP1* (95 %), *KIR2DS4* (92 %) and *KIR3DL1* (93 %).

A recent study of the association between polymorphisms in *KIR* and *HLA* genes and pediatric ALL in Hispanic and non-Hispanic children provided additional evidence about the contribution of genetic variation in ALL incidence. When the incidence and survival were evaluated between the two ethnic groups, a high incidence of ALL and a significantly worse survival was found in Hispanic children compared to non-Hispanic Whites. The genotypes diversity related to KIR and HLA ligands are very suggestive that these two loci may determine a different susceptibility effect depending on the ethnic groups. Such observed differences are probably multifactorial due to an interaction between KIR and environmental factors, e.g. patterns of infection, rather than merely allele frequencies differences between ethnic groups [14].

In a study carried out in Italian population Bontadini and colleagues reported the same general *KIR* gene patterns distribution observed in other Caucasian and non-Caucasian populations. Australian Aborigine, Chinese Han, and Japanese showed the most markedly different patterns, with significant differences from Italian population and other Caucasian populations, in particular for inhibitory gene *KIR2DL2* and non inhibitorys *KIR2DS1*, *KIR2DS2*, *KIR2DS3*, *KIR3DS1* [17–20]. The findings with respect to *KIR* gene diversity in different populations could provide relevant genomic diversity data for further studies on viral infection, autoimmune diseases, and reproductive fitness.

Inhibitorys genes *KIR2DL2* and *KIR2DL5B* were also found at lower frequencies in the Italian population, as well as the activating genes *KIR2DS3* and *KIR2DS4* [15]. Notably, as to *KIR2DS4* alleles, Han Chinese showed an inverse pattern compared to the Italian population [19].

The activating KIR2DS4 gene is unique in the haplotype A, whereas haplotype B contains up to five activating KIRs. Haplotypes A and B have been preserved in the human population (about 25 and 75 % in Caucasian), thus suggesting the occurrence of a balancing selection [13, 15].

Linkage disequilibrium or the non-random associations between alleles at two loci are also present in KIR genes repertoire. A high positive linkage disequilibrium between *KIR2DL1* and *KIR2DL3* has been observed in Caucasian and non-Caucasian populations [21]. Our data are consistent with this hypothesis since the frequencies of these two genes were the highest observed in both control and patient groups.

**Fig. 1:**
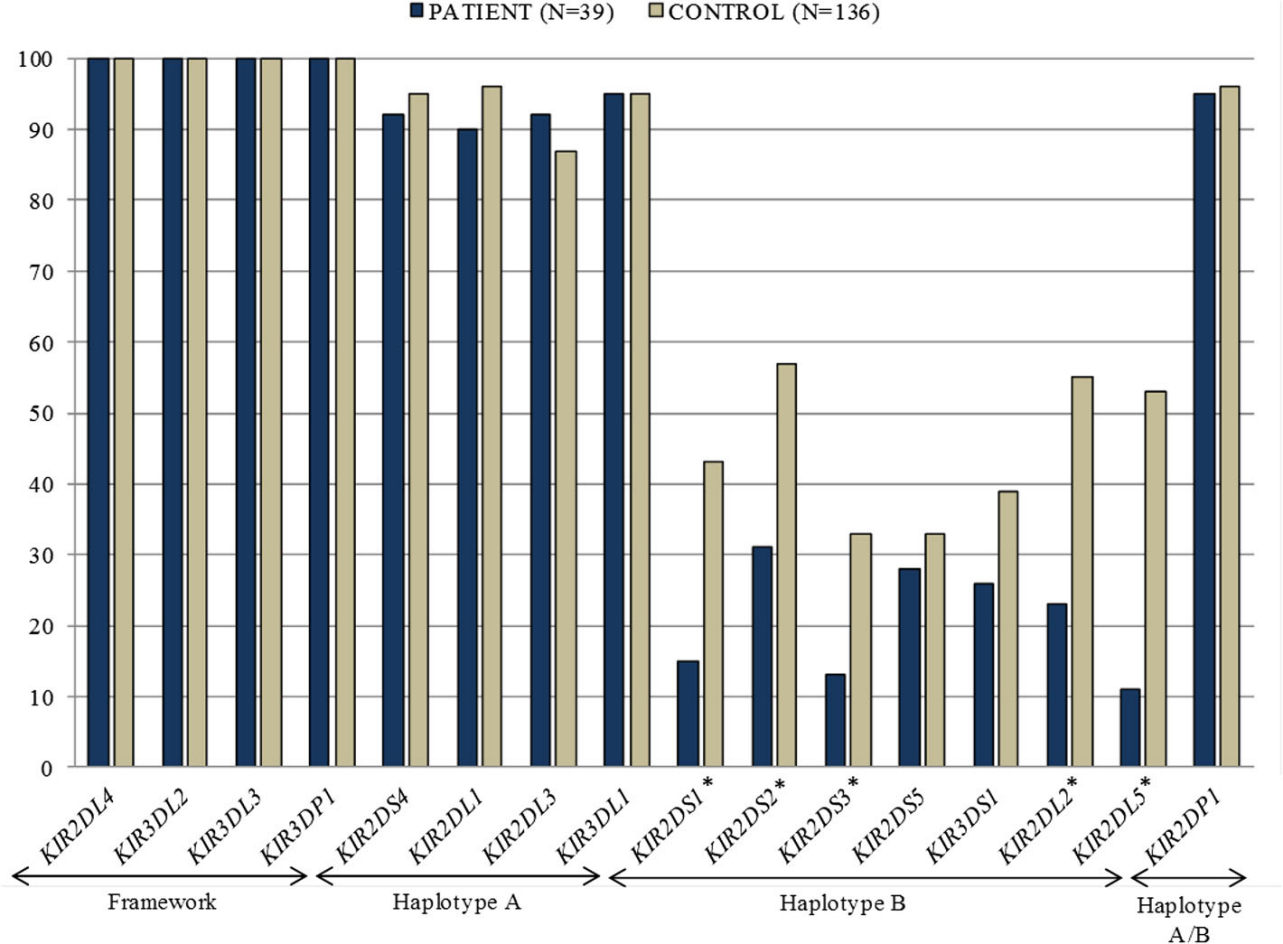
Phenotypic frequency related to the presence/absence of *KIR* genes in patients (*N* = 39) and healthy controls (*N* = 136). Genes marked with (*) showed significance for the stastical test

*KIR r*epertoire comparisons between patients and healthy family members (Fig. 1) showed that the inhibitory genes *KIR2DL2* (*p* = 0.0005) and *KIR2DL5* (*p* = 0.0067), as well as the activating genes *KIR2DS1* (*p* = 0.0013), *KIR2DS2* (*p* = 0.0038), *KIR2DS3* (*p* = 0.0153) were more frequently found (*p* <0.05) in healthy individuals than in patients.

HLA-KIR genotypes have been associated with susceptibility to a variety of diseases such as psoriatic arthritis, type I diabetes, infectious diseases, cancer, and reproduction. Just a few of these studies revealed an influence of HLA-KIR gene interactions on disease outcome [9]. Others studies have investigated the frequency of KIR genes in patients with hematologic malignancies [22]. Most of these investigations have been performed in patients with different diseases such as AML, CML and MDS [23–27].

There are several studies that investigate the association between *KIR* haplotypes distribution and diseases; it is observed that Haplogroups A and B vary considerably between ethnic groups [15, 28–30]. The association of *KIR* gene/haplotypes has been investigated in patients with indication for hematopoietic stem cell transplantation, highlighting the role of *KIR* genes in the transplant outcome [7].

**Fig. 2:**
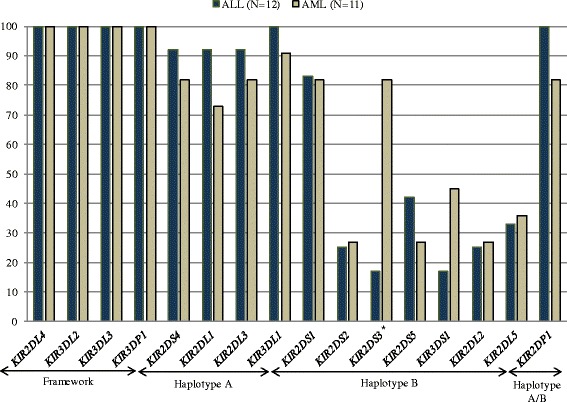
Phenotypic frequency related to the presence/absence of *KIR* genes in ALL patients (*N* = 12) and AML patients (*N* = 11). Genes marked with (*) showed significance for the stastical test

The stratification of the patients according to AML *vs* ALL groups, revealed that the *KIR2DS3* gene presented higher frequencies (*p* = 0.0031) in AML when compared to ALL patients (Fig. 2). Although the sample size was relatively small, recent publications suggest important roles for specific KIR genes that may influence allogeneic hematopoietic stem cell transplantation (HSCT) outcome in HLA-compatible siblings, GvHD, relapse and other complications related to transplantation [31] in small and heterogeneous samples [32].

A study in China investigated KIR genotypes in 54 patients with hematopoietic malignancies, classified into two risk groups: standard and high. The frequency of activating *KIR* genes in standard-risk group was higher when compared to the high-risk group, specifically for *KIR2DS1*, *KIR2DS2* and *KIR3DS1*. A secondary analysis of this study, comparing standard-risk group *vs* high-risk group in AML patients, revealed higher frequencies of activating *KIR* genes in the standard-risk group, particularly for *KIR2DS1, KIR2DS2,* and *KIR2DS3* genes, the latter one in agreement with our findings in AML patients group [22].

In the same line of investigation, Kim and colleagues reported the influence of *KIR* genes in AML patients and HLA compatible donor siblings after HSCT. All the activating *KIR* genes in the donors showed an important role in transplant outcome and in the occurrence of acute graft-versus-host disease (GvHD) in HSCT in AML patients. Particularly, the *KIR2DS2* gene and the allele KIR2DS4*003 were correlated with acute GvHD. This evidence suggests an immunogenic specificity in the Korean population compared to Caucasians since the frequency of *KIR2DL2* and *KIR2DS2* genes are comparatively lower in Koreans than in other countries. Long-term survival was noted even if the KIR2DS1 gene was only present in the donor and not in the recipient. The presence of both genes KIR2DS3-KIR2DS5 was more frequently found in a variety of complications related to transplant [31].

Mancusi and colleagues reported that donors, possessing KIR2DS1, KIR3DS1 or both activating genes, showed reduced infection rates and mortality, and a better event-free survival (EFS) [33].

Donor cells that express KIR haplotype B have been reported to contribute to relapse protection and improved survival after myeloablative allogeneic transplantation. Haplotype B/x donor cells have also been associated with a higher incidence of chronic GvHD. In HLA-haploidentical transplant setting, adonor *KIR* B haplotype has been associated with lower risk of relapse for patients with hematologic malignancies [2].

NK cells receptors of the family KIR may confer specific protector effect for different diseases. In a Turkish study carried out in a heterogeneous group of leukemia patients and controls, a protective effect was observed associated with KIR2DL2 and/or KIR2DS2 against CML [32].

From an evolutionary perspective, activating *KIR*s arose more recently from inhibitorys homologous genes [34]. A wide variation in *KIR* activators gene frequencies have been reported for different populational groups [35]. Nevertheless, the allelic diversity related to inhibitory receptors genes is limited when compared to activators genes [36]. The strong negative correlation observed between certain activating KIR and its ligands across populations, in contrast to weak positive correlations between several KIR inhibitory genes and their ligands, put forward a hypothesis that a pressure selection mechanism involving autoimmune disease is acting on the maintenance of lower frequencies of activator KIR receptor and their ligands [35].

KIR phenotypes analysis in Belgian leukemia patients indicated significantly higher frequencies of inhibitory *KIR2DL1, KIR2DL2,* and *KIR2DL3* genes, suggesting their contribution for the lack of antitumor responses of NK cells [10]. On another study, conducted in 35 patients with a lymphoproliferative NK cell disease, inhibitory genes *KIR2DL5A* and *KIR2DL5B* were more frequently found in patients compared to healthy controls [37].

Epstein-Barr virus has been associated to the development of Hodgkin’s disease in some pathological conditions. An important review summarises current knowledge of the pathogenesis of Hodgkin’s disease with particular emphasis on the association with EBV. Besson and colleagues identified in a family study a stronger protector effect related to *KIR2DS1/KIR3DS1* in patients with Hodgkin’s lymphoma [38].

A total of 50 Han Chinese patients were studied to explore the correlation between *KIR* genes and susceptibility to leukemia. The comparison made between patient and control groups showed lower frequencies of *KIR3DL1* and *KIR2DL1* genes amongst patients. Additionally, the results highlighted a negative correlation between the pathogenesis of leukemia and *KIR3DL1*, *KIR3DS1, KIR2DL1,* and *KIR2DL5* genes [39], a very suggestive finding that *KIR* polymorphisms are associated with susceptibility to leukemia in Hans.

In the present study, patients had lower frequencies of *KIR3DS1* (26 % versus 39 %) and *KIR2DL5* (28 % versus 53 %) as compared to healthy family members group.

McQueen and collaborators analyzed *KIR* genes repertoire in donors, and found that *KIR2DS3* conferred a protective effect against chronic GvHD in transplantation with HLA-compatible unrelated donor [40] and with donors who have more than four activating KIR in haploidentical transplants.

NK cells were components previously not recognized in HSCT rejection process and GvHD. More recently, investigation of human health/disease effects associated with KIR receptors have been reported and the majority of the described associations have been with activators *KIR* genes. Our data suggest that susceptibility to leukemia can be influenced, at least, partly by KIR receptors and an increased sample can confirm these findings for further investigations.

## Conclusion

Our study with KIR receptors in patients with hematologic diseases showed that inhibitory genes *KIR2DL2* and *KIR2DL5* and activating genes *KIR2DS1*, *KIR2DS2*, *KIR2DS3* were more frequently found in healthy family members than in patients. Also, the *KIR2DS3* was significantly more frequent in patients with AML when compared to patients with ALL. We observed a higher frequency of haplotype A in the patients group. All these data are crucial to understanding the mechanisms underlying the dysfunction of NK in leukemia. The characterization of the genetic profile of patients brings together relevant information to enable robust investigations of *KIR* genes and their influence on important diseases such as Acute Myeloid Leukemia, Acute Lymphoblastic Leukemia, and others.

## Additional files

Additional file 1: Patients – Supporting Information. (XLS 43 kb) Additional file 2: Healthy Family Members – Supporting Information. (XLS 69 kb)

## Abbreviations

ALL: Acute lymphoblastic leukemia
AML: Acute myeloid leukemia
BMT: Bone marrow transplantation
CML: Chronic myeloid leucemia
DNTPs: Deoxynucleotide
EDTA: Ethylene tetraacetic acid
EFS: Event-free survival
GvHD: Graft-versus-host disease
HCT: Hematopoietic cell transplantation
HLA: Human leukocyte antigen
HSCT: Hematopoietic stem cell transplantation
IBGE: InstitutoBrasileiro de Geografia e Estatística
ILTs: Immunoglobulin-like transcript
*KIR*: Immunoglobulin-like receptors of natural killer
LCR: Leukocyte receptor complex
LIGH: Laboratório de imunogenética e histocompatibilidade
MDS: Myelodysplasia
MHC: Major histocompatibility complex
NK: Natural killer
PCR-SSOP: Polymerase chain reaction - sequence specific oligonucleotide probes
